# Normative data for Vietnamese population: Effects of age, education, and sex on test performance

**DOI:** 10.1017/S1355617725101100

**Published:** 2025-08

**Authors:** Truc Tran Thanh Nguyen, Thanh-Liem Do, Huong Thi Thu Tran, Ingo Kilimann, Cong-Thang Tran

**Affiliations:** 1 Memory and Dementia Center, Hospital 30/4, Ho, Chi Minh City, Vietnam; 2 Taiwan International Graduate Program in Interdisciplinary Neuroscience, National Taiwan University and Academia Sinica, Taipei, Taiwan; 3 Deutsches Zentrum für Neurodegenerative Erkrankungen (DZNE), site Rostock/Greifswald, Rostock, Germany; 4 Department of Psychosomatics, Rostock University Hospital, Rostock, Germany; 5 Department of Neurology, University of Medicine and Pharmacy at Ho Chi Minh City, Ho Chi Minh City, Vietnam; 6 Memory and Dementia Center, University Medical Center at Ho Chi Minh City, Ho Chi Minh City, Vietnam

**Keywords:** Norms, Vietnam, neuropsychology, cognition, education, aging

## Abstract

**Objective:**

Normative data of neuropsychological tests in the Vietnamese population is considerably lacking. We aim to evaluate the effects of age, education, and sex on the performance of common neuropsychological tests, and to generate normative data for these tests in cognitively normal Vietnamese adults.

**Method:**

Participants were recruited from two hospitals in Ho Chi Minh City, with inclusion criteria as follows: age ≥ 40 years, normal cognition and function, and Mini-Mental State Examination (MMSE) scores ≥ 26. Neuropsychological tests were administered in a paper-and-pencil format, including the CERAD Word List, Trail Making Tests, Digit Span, Animal Naming, and Clock Drawing Test. Effects of age, education, and sex on test performance were evaluated using multiple linear regression analyses. Normed scores were reported as regression-based and discrete norms tables.

**Results:**

Participants included 385 cognitively normal Vietnamese, with age 61.4 ± 10.9 years (range 40 – 89), female 56%, who were relatively highly educated (42% attended college and beyond, 36% attended high school or equivalent institutions, 22% had less than high school education), and had MMSE scores 27.8 ± 1.0. Trail Making Test Part B was completed within 300 s by only 204/385 (53%) participants. Regression analyses demonstrated significant associations between age and education with performance on all or most tests, and between sex and all CERAD Word List measures and Clock Drawing Test.

**Conclusions:**

The present work provides the first known normative data for a relatively comprehensive neuropsychological battery in Vietnamese adults. Performance on all tests was significantly influenced by age and education.

## Statement of Research Significance

**Research Topic(s):**

- Neuropsychological testing is the evaluation of cognitive abilities. An individual’s scores on neuropsychological tests have to be benchmarked against those of well-performing, cognitively normal people. Our goal was to develop normative data for a comprehensive neuropsychological battery for Vietnamese adults and assess the influence of demographic characteristics on test performance.

**Main Findings:**

- We found age and education to be consistently and significantly associated with cognitive ability, such that people who are younger or more educated performed better on most or all tests. A sex effect was found in tests of memory and visuospatial function. Normative data are provided as tables and equations so that they can be readily applied in clinical settings.

**Study Contributions:**

- We generated normative values for commonly used neuropsychological tests and examined the influence of age, sex, and education on test performance in nearly 400 Vietnamese cognitively healthy adults, age 40 and above.

## Introduction

Neuropsychological examination is an integral part of the comprehensive evaluation of brain functions. Neuropsychological reports can determine areas of strength and weakness in cognitive domains related to a wide range of neuropsychiatric disorders. It is particularly helpful in determining the presence and severity of dementia, cerebrovascular diseases, and post-traumatic brain injury (TBI) or concussions, all of which are among the fastest-growing causes of disability in Vietnam (Carr et al., 2018). For instance, Vietnam ranked #21 among countries with the highest number of older adults in 2020 (https://www.prb.org/), leading to a corresponding rapid increase in the prevalence of dementia (Vu et al., 2024). Stroke incidence of Vietnam is among the world’s highest, at about 161 per 100000 (Mai et al., 2022); while the incidence of TBI is at 291 per 100000, representing a 50.4% increase from 1990 to 2016 (James et al., 2019).

To interpret the results, scores from various cognitive tests must be benchmarked against normative data – a frame of reference considered to be “normal” performance generated by a cohort of cognitively healthy individuals (Mitrushina et al., 2005). Such normative values, while can be found for many neuropsychological tests among American and European individuals, remain scant in less developed countries, including Vietnam, primarily due to the challenging nature of conducting normative research (e.g., adequate sample size and funding are required). It is now well established that performance in neuropsychological tests is considerably influenced by age, sex, education, and cultural factors, and that tests of Western origins can potentially misclassify individuals from non-Western populations, ethnic minorities, or with disadvantageous socioeconomic background (Fillenbaum et al., 2005; Franzen et al., 2020; Nguyen et al., 2024). In addition, comprehensive neuropsychological evaluation in Vietnam is sadly lacking, with no formal training or certification in neuropsychology being provided nationwide as of present. Therefore, it is important to generate normative performance from an adequately powered cohort to improve diagnostic accuracy and allow early detection of mild cognitive impairment (MCI) and dementia among Vietnamese individuals.

Previous research to generate normative values that account for sex, age, and educational background in the Vietnamese-speaking population was conducted more than 10 to 20 years ago, largely in participants residing in the United States (Dick et al., 2002; Ngo et al., 2010), with small sample size (*N* = 30 – 60), or using primarily a single measure (e.g., verbal fluency in (Kempler et al., 1998; Strauss et al., 2006) or Stroop task in (Doan & Swerdlow, 1999). At the present time, no normative data of neuropsychological tests in Vietnam have been published. Therefore, our study aims to extend prior efforts by developing a relatively comprehensive neuropsychological battery, assessing the influence of demographic variables of interest on test performance, and establishing updated normative data to inform clinical decision making for Vietnamese adults (McCauley et al., 2023).

## Method

### Participants

Participants were recruited from two medical centers, Hospital 30/4 (read “April 30”) and University Medical Center (UMC), in Ho Chi Minh city in southern Vietnam. Hospital 30/4 is a central hospital and is part of the healthcare system operated by the Ministry of Public Security. It primarily serves active and retired police officers and their families, although the general public can also seek and receive care. UMC is a university hospital, affiliated with the University of Medicine and Pharmacy at Ho Chi Minh city. Both are general hospitals with a neurology department and memory and dementia unit.

Participants were (i) individuals seeking pre-employment, employee health checkup, or general physical exams; (ii) spouses of people with dementia or MCI visiting the memory and dementia unit; or (iii) visitors of inpatients. Halfway through the data collection, it was found that adults over the age of 80 were significantly underrepresented in the study, hence recruitment efforts were also conducted at a community center where senior social clubs hold weekly gatherings in Ho Chi Minh city (in the same district as the two aforementioned hospitals). We included participants who met the following criteria: (i) age ≥ 40 years, (ii) self-identified as having normal cognition and daily function when interviewed (i.e., denied any cognitive complaints; able to perform basic and instrumental activities of daily living without assistance), (iii) Mini-Mental State Examination (MMSE) scores ≥ 26 (Folstein et al., 1975; Kvitting et al., 2019), and (iv) could read and write. Participants who acknowledged a history of neuropsychological disorders, psychiatric illness (including schizophrenia and bipolar disorder), substance abuse, head injury with loss of consciousness, or having visual or hearing impairment were excluded. Sample size was calculated based on recommendations of at least 50 participants per age group for normative studies (Mitrushina et al., 2005), totaling 250 participants for five age groups (40–49, 50–59, 60–69, 70–79, and 80–89).

Data on the demographic characteristics (age, sex, education; with education recorded as less than high school, high school or equivalent, or college and beyond) and history of any chronic illnesses (e.g., cardiovascular disease, diabetes, cancer) of the participants were collected. All participants provided written informed consent. The research was approved by the Institutional Review Board of the University of Medicine and Pharmacy at Ho Chi Minh City (Approval No. 615/HDDD-DHYD on September 24 2020). The study was completed in accordance with the Helsinki Declaration. Data were collected between March 2021 and October 2023.

### Test selection for the neuropsychological battery

The neuropsychological tests were selected based on the following three criteria. First, they should encompass a broad range of cognitive domains that are relevant to the diagnosis of cognitive impairment: memory, attention, executive function, processing speed, language, and visuospatial ability. Second, we opted for instruments that are well-established and commonly used (Rabin et al., 2005) rather than developing new tests, as this would enable meaningful comparison with other cross-cultural studies. Finally, test administration should be completed within 30 minutes to facilitate implementation in various clinical settings, including low- to medium-resource facilities. As a result, the following tests were chosen: Consortium to Establish a Registry for Alzheimer’s Disease (CERAD) Word List (WL) Tasks, Trail Making Test (TMT), Digit Span, Animal Fluency, and Clock Drawing Test.

### Neuropsychological measures

As there are currently no neuropsychologists in Vietnam, neuropsychological tests were administered by nurses who were trained under the guidance of the study’s corresponding author (CTT), with all testing sessions conducted under the supervision of a Ph.D.-level neurologist (author HTTT). Tests were administered using a paper and pencil format, including:

*CERAD WL Tasks* (Morris et al., 1989), consisting of the WL Memory, WL Recall, and WL Recognition. The WL Memory is a free immediate recall task of 10 printed words over three trials, with the 10 words presented in a new random order in trials 2 and 3. WL Recall and WL Recognition test for delayed recall and recognition of the 10 words, respectively. Permission to translate and adapt the CERAD WL Tasks into Vietnamese was granted by Dr Gerda Fillenbaum (personal email communication, February 1, 2020). We opted to employ the high frequency concept during translation, i.e., choosing words referring to objects familiar to the Vietnamese population rather than using word-for-word translation from the original CERAD WL. The original WL was constructed by selecting common English nouns, of one to two syllables, with a frequency of ≥ 100/million or 50-99/million words from the Thorndike-Lorge list (Morris et al., 1989; Thorndike & Lorge, 1944). Following this conceptualization, we developed the Vietnamese version of CERAD WL with words that are Vietnamese nouns commonly used in daily life and reported to have high frequency of usage based on Google search engine (i.e., words that occur ≥ 10,000 times using the Google search engine for Vietnamese words). We conducted a pilot testing on a small sample of cognitively unimpaired individuals who sought health checkup at Hospital 30/4 to confirm that the Vietnamese words were indeed frequently used (e.g., to assess the Vietnamese word “head,” individuals were asked if the statement “The head is part of the human body” was true or false). Finally, a copy of the Vietnamese translation, including the English equivalent for each word, was sent to Dr Fillenbaum for review. Please refer to Supplementary Methods and Supplementary Table S1 for the Vietnamese version of WL Tasks. We have previously demonstrated that the Memory, Recall, and Recognition tasks all have high test-retest reliabilities with *r*s of 0.81, 0,86, 0.77, respectively (all *p*-values < .05) (Nguyen & Tran, 2022). Following the administration of WL Memory, a gap of 10 to 15 minutes took place before the WL Recall and WL Recognition were given, during which we administered the TMT, Parts A and B.

*TMT, Parts A (TMT-A) and B (TMT-B)* (Reitan & Wolfson, 1993). The TMT measures attention, psychomotor speed, and mental flexibility. TMT-A requires participants to draw lines that consecutively connect 25 encircled numbers on a sheet of paper. TMT-B has a similar requirement except that one has to alternate between numbers and letters. Participants are allowed 180 s to complete Part A and 300 s to complete Part B. Variables of interest are time to completion on each part in seconds. If participants have not completed the Trail by the time limit, timing is stopped and scores are not recorded (though participants are allowed to progress if they are likely to finish soon as judged by the examiner (Heaton et al., 2004). Our rationale for not assigning the maximum TMT-A/TMT-B score of 180/300 for participants who do not complete the task, as is conventionally done (Strauss et al., 2006), is that this practice potentially diminishes performance variability among people with varying levels of executive dysfunction (Correia et al., 2015) and leads to violation of the linear regression assumptions to calculate norms in our cohort (see Results). While a few studies have included all TMT-B completion times rather than a maximum of 300 s (MacPherson et al., 2017), we opted to discontinue the task to minimize participants’ frustration, reduce testing time, and to conform with most standard administration instructions (Bowie & Harvey, 2006; Strauss et al., 2006). Additionally, in TMT-B, we kept the letters as English alphabet (A B C D E …) instead of changing them into Vietnamese alphabet (A Ă Â B C D Đ E Ê …) as we anticipated that the participants would be familiar with the Latin alphabet.

*Digit Span* (Wechsler, 2008), consisting of Digit Span Forward and Digit Span Backward, which are measures of attention and working memory, respectively. In the Digit Span Forward, participants are read sequences of numbers of increasing lengths and asked to repeat them in the same order. In the Digit Span Backward, participants are also read sequences of numbers of increasing lengths but are asked to repeat each sequence in reverse order. Variables of interest are the longest correct number of sequential digits.

*Animal Fluency* (Kertesz, 1982), a test of free recall that evaluates verbal category fluency and semantic memory among other abilities, including executive functioning, attention, and speed of processing. Participants are asked to name as many animals as they can in 60 s. Variable of interest is the total word count.

*Clock Drawing Test* (Shulman et al., 1986), which is commonly used to assess executive function, visuospatial organization, and abstract thinking. Participants are shown a pre-drawn circular contour, then asked to draw numbers within the circle to make it look like a clock. As there is no Vietnamese word-for-word equivalent of the English “10 after 11” or “10 past 11,” participants were instructed to set the time “at 11:10.” Possible scores range from 1 to 6 based on the modified Shulman’s scoring system, given as followed: 1 = perfect, 2 = minor visuospatial errors (e.g., mildly impaired spacing of times); 3 = inaccurate representation of 10 after 11 when visuospatial organization is perfect or shows only minor deviations (e.g., minute hand points to 10); 4 = moderate visuospatial disorganization of times such that accurate denotation of 10 after 11 is impossible; 5 = severe level of disorganization as with 4 points; and 6 = no reasonable representation of a clock.

Higher scores indicate better performance on all tests, except for TMT and Clock Drawing Test, for which a higher score indicates longer time to completion or more clock-drawing errors, respectively, and therefore worse performance. The manuscript reporting the validity of these tests for the Vietnamese population is currently in preparation.

### Statistical analysis

Descriptive statistics are provided with continuous variables presented as mean and standard deviation, while categorical variables are presented as frequencies and proportions. Normative data were generated via two approaches, the traditional discrete norms and multiple regression equations. Using the first approach, normative data for each neuropsychological test are provided as statistics of raw test scores stratified by age, education, and sex, if the latter showed significant effect on test performance. For each demographic group, we report the mean, standard deviation, and test scores at the 3^rd^ (–2 SD), 7.5^th^ (–1.5 SD), 15^th^ (–1 SD), 25^th^, 50^th^, 75^th^, and 95^th^ percentiles. The –1.5 and –1 SD are presented because these have been used as cutoff values in neuropsychological criteria to diagnose mild neurocognitive disorder; the –2 SD is the cutoff value to determine the presence of major neurocognitive disorder (American Psychiatric Association, 2013).

In the second approach, hierarchical linear regression analyses were conducted with cognitive test scores as dependent variables and age, education, and sex as independent variables. Reference groups are education < high school and male. Education was entered first into the regression equations, followed by age and sex, as education has been documented to have the greatest influence on test performance (Dick et al., 2002). If any of the three independent variables was found to have non-significant coefficient, it was dropped from the model, and the model was rerun with the remaining variables. We also calculated the proportion of variance (eta^2^) accounted for by education, age, and sex in each cognitive test. All analyses were performed using R version 4.2.0.

## Results

### Participant characteristics

Participant demographic, clinical, and cognitive characteristics are shown in Table 1. Table 2 shows the number of participants stratified by age group, level of education, and sex . Overall, a total of 385 participants were included in this study, with mean age of 61.4 (*SD* = 10.9, range 40–89). More than half of them were female (57.1%). Participants were highly educated, with nearly half having completed college or a higher degree. Hypertension was the most common comorbidity of the cohort (39.7%), followed by dyslipidemia (28.5%) and diabetes mellitus (14.5%). Participants needed on average 28 minutes (–89). More than half of them were female (57.1%). Participants were highly educated, with nearly half having completed college or a higher degree. Hypertension was the most common comorbidity of the cohort (39.7%), followed by dyslipidemia (28.5%) and diabetes mellitus (14.5%). Participants needed on average 28 minutes (*SD* = 6.7) to complete the neuropsychological battery. Data for all neuropsychological tests were mostly available (rate of missing data varied between 0.5% and 2.5%), except for TMT-B, where only 53% of the cohort were able to complete the test within the time limit. The remaining participants either did not complete TMT-B within 300 s (136/385 individuals, 35.3%) or could not complete it (43/385, 11.1%) due to several reasons, including not understanding test instructions or inability to recall the sequence of the English alphabet. Compared to those who completed TMT-B within the time limit, non-completers were significantly older, less educated, and demonstrated poorer performance in measures of memory, attention, executive, and visuospatial function, but not language (all *p*-values < .001, Supplementary Table S2). Assigning the maximum score of 300 to participants who could not complete within 5 minutes (Strauss et al., 2006; Weintraub et al., 2018) leads to heavily skewed distribution of TMT-B scores and violation of homoscedasticity assumption of the linear regression model to calculate continuous norms, hence the remaining analyses for TMT-B scores were conducted using data of participants who completed this test within the allotted time.

**Table 1.** Participant characteristics

Characteristic	Total (*N* = 385)
***Demographic***	
Age, *M* (*SD*)	61.4 (10.9)
Age group, *n* (%)	
40–49	70 (18.18)
50–59	90 (23.37)
60–69	129 (33.50)
70–79	73 (18.96)
80–89	23 (5.99)
Education, *n* (%)	
Less than high school	85 (22.07)
High school or equivalent	138 (35.84)
College and beyond	162 (42.09)
Female, *n* (%)	220 (57.14)
***Clinical,*** *n* (%)	
Hypertension	153 (39.74)
Diabetes mellitus	56 (14.54)
Dyslipidemia	110 (28.57)
***Neuropsychological***, *M* (*SD*)	
MMSE (26–30)	27.81 (1.09)
CERAD WL memory (9–30)	21.14 (4.24)
CERAD WL recall (0–10)	7.10 (1.82)
CERAD WL recognition (3–10)	9.02 (1.37)
Trail making test, Part A^a^ (15–180)	69.58 (31.53)
Trail making test, Part B^b^ (27–300)	152.06 (63.64)
Digit span forward (0–12)	8.86 (2.11)
Digit span backward (2–12)	5.73 (2.27)
Animal naming test^c^ (8–30)	19.01 (4.63)
Clock drawing test^d^ (1–5)	1.66 (0.81)
Neuropsychological test administration time, minutes^e^ (10–80)	28.46 (6.72)

*Note*: CERAD WL = Consortium to Establish a Registry for Alzheimer’s Disease Word List Tasks, MMSE = Mini-Mental State Examination. Data on continuous variables are presented as *M* (*SD*); data on categorical variables are presented as *n* (%). Min and max scores of neuropsychological data are presented in parentheses next to the neuropsychological entries.

a Missing in 10/385 participants (2.5%).

b Missing in 181/385 participants (47.0%).

c Missing in 2/385 participants (0.5%).

d Missing in 3/385 participants (0.7%).

e Missing in 5/385 participants (1.2%).

**Table 2.** Number of participants stratified by sex, age, and education

Age	Level of education								
	< High school			High school & equivalent			College and beyond		
	Female	Male	Total	Female	Male	Total	Female	Male	Total
40–49	5	5	10	12	4	16	26	18	44
50–59	13	6	19	30	11	41	16	14	30
60–69	21	7	28	36	14	50	25	26	51
70–79	8	11	19	9	16	25	8	21	29
≥80	5	4	9	2	4	6	4	4	8

### Discrete normative data

Given the small sample size of cells where participants were further broken down by sex, we subsequently provided discrete norms tables stratified by age group and education only. Summary statistics (mean, SD) of all neuropsychological scores are provided in Table 3, while discrete norms by age and education, including performance at the 3^rd^, 7.5^th^ (–1.5 SD), 15^th^ (–1 SD), 25^th^, 50^th^, 75^th^, and 95^th^ percentiles, are provided in Supplementary Tables S3–S11. As an example, for a 71-year-old male with ≥ college education, a CERAD WL Memory score of 14 would correspond to performance at the 7.5^th^ percentile (Supplementary Table S3).

**Table 3.** Discrete norms reported as mean and SD by age group and level of education

	Age	Level of education		
		< High school	High school	College +
CERAD WL memory	40–49	23.70 (4.37)	22.75 (3.82)	24.15 (3.41)
	50–59	19.42 (4.11)	22.53 (2.96)	22.73 (4.43)
	60–69	20.03 (3.24)	21.62 (4.18)	21.62 (3.81)
	70–79	17.26 (3.64)	18.08 (4.13)	19.72 (3.70)
	80–89	17.00 (2.44)	17.33 (4.67)	18.00 (2.44)
CERAD WL recall	40–49	8.30 (1.41)	7.93 (0.99)	8.50 (1.35)
	50–59	6.42 (1.83)	7.36 (1.98)	7.63 (1.24)
	60–69	6.71 (1.90)	7.06 (1.81)	7.11 (1.59)
	70–79	6.63 (1.73)	6.12 (1.64)	6.51 (1.80)
	80–89	5.33 (1.80)	5.00 (2.00)	5.62 (1.30)
CERAD WL recognition	40–49	9.60 (0.69)	9.56 (0.62)	9.68 (0.70)
	50–59	9.00 (1.05)	9.36 (0.88)	9.13 (1.38)
	60–69	8.64 (1.72)	9.10 (1.47)	9.00 (1.11)
	70–79	8.68 (1.66)	8.04 (1.79)	8.75 (1.35)
	80–89	8.22 (2.16)	7.83 (2.63)	9.12 (0.64)
Trail making test, Part A	40–49	74.20 (31.77)	67.00 (25.37)	42.50 (12.59)
	50–59	74.00 (38.51)	68.00 (25.63)	50.20 (15.02)
	60–69	77.50 (36.05)	72.02 (27.48)	66.62 (24.60)
	70–79	83.66 (27.25)	92.00 (38.94)	67.68 (25.88)
	80–89	143.85 (20.36)	107.80 (42.91)	87.71 (19.17)
Trail making test, Part B	40–49	202.80 (60.73)	169.75 (65.30)	108.57 (49.21)
	50–59	187.00 (35.25)	158.16 (70.38)	132.66 (52.73)
	60–69	131.75 (21.62)	171.73 (55.54)	147.77 (64.61)
	70–79	174.50 (7.77)	141.00 (48.28)	190.45 (60.12)
	80–89	298.00 (NA)^a^	221.00 (15.55)	186.66 (83.26)
Digit span forward	40–49	9.10 (2.23)	9.06 (2.97)	10.18 (1.67)
	50–59	9.05 (1.89)	9.29 (1.84)	9.43 (1.69)
	60–69	7.57 (1.89)	8.80 (2.11)	9.35 (1.80)
	70–79	7.21 (2.48)	7.60 (2.04)	8.17 (1.98)
	80–89	8.66 (1.73)	8.50 (2.07)	8.62 (1.68)
Digit span backward	40–49	5.50 (2.27)	5.50 (1.26)	7.09 (2.52)
	50–59	4.52 (1.74)	6.24 (2.59)	6.53 (2.50)
	60–69	4.96 (1.52)	5.78 (2.20)	6.25 (2.38)
	70–79	4.57 (1.57)	4.80 (1.87)	5.62 (1.71)
	80–89	4.11 (1.36)	5.50 (2.34)	3.37 (0.91)
Animal naming test	40–49	19.20 (4.23)	20.12 (4.51)	22.44 (3.91)
	50–59	16.78 (4.25)	19.14 (4.07)	20.43 (4.32)
	60–69	17.50 (4.79)	18.34 (4.51)	20.03 (4.56)
	70–79	16.88 (3.81)	17.76 (4.64)	18.41 (5.19)
	80–89	15.88 (3.88)	17.00 (3.94)	16.00 (2.50)
Clock drawing test	40–49	1.30 (0.48)	1.43 (0.72)	1.40 (0.49)
	50–59	2.05 (0.87)	1.65 (0.69)	1.33 (0.47)
	60–69	1.77 (0.89)	1.74 (0.87)	1.50 (0.64)
	70–79	1.77 (0.80)	2.00 (1.19)	1.44 (0.57)
	80–89	2.88 (1.26)	2.16 (1.16)	2.12 (0.83)

*Note*: CERAD WL = Consortium to Establish a Registry for Alzheimer’s Disease Word List. Data were presented as mean (standard deviation).

a The standard deviation of Trail Making Test, Part B for individuals aged 80–89 with less than high school education was not available as there was only one participant in this demographic.

### Regression-based norms

Regression-based demographically corrected norms can be calculated from Table 4 using the regression coefficients and Root Mean Squared Error (RMSE) . Using the previous example, to obtain the expected performance (i.e., 50^th^ or at *z*-score = 0) on CERAD WL Memory for a 71-year-old male with ≥ college education:

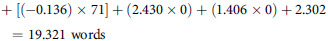




**Table 4.** Linear regression analyses for demographic variables and neuropsychological test performance

Neuropsychological tests	Intercept	Age	Female	High school	College	RMSE	*F* test statistics
CERAD WL memory	26.675	−0.136***	2.430***	1.406**	2.302***	3.553	*R*^*2*^ = .29, *F*(4, 380) = 39.79, *p* < .001
CERAD WL recall	9.910	−0.058***	0.845***	n.s.	0.580*	1.597	*R*^*2*^ = .22, *F*(4, 380) = 28.31, *p* < .001
CERAD WL recognition	10.645	−0.030***	0.401*	n.s.	n.s.	1.304	*R*^*2*^ = .08, *F*(2, 382) = 19.24, *p* < .001
TMT-A^a^	17.035	1.043***	n.s.	n.s.	−21.073***	27.546	*R*^*2*^ = .22, *F*(3, 371) = 37.89, *p* < .001
TMT-B^a, b^	76.101	1.849***	n.s.	n.s.	−42.821**	58.380	*R*^*2*^ = .14, *F*(3, 200) = 12.15, *p* < .001
Digit span forward	11.060	−0.045***	n.s.	n.s.	1.023***	1.994	*R*^*2*^ = .09, *F*(3, 381) = 15.07, *p* < .001
Digit span backward	7.479	−0.042***	n.s.	0.843**	1.335***	2.144	*R*^*2*^ = .09, *F*(3, 381) = 15.15, *p* < .001
Animal naming test	24.346	−0.110***	n.s.	1.138*	2.550***	4.310	*R*^*2*^ = .12, *F*(3, 379) = 19.30, *p* < .001
Clock drawing test^a^	0.860	0.014***	0.168*	n.s.	−0.346**	0.775	*R*^*2*^ = .07, *F*(4, 377) = 8.99, *p* < .001

*Note*: CERAD WL = Consortium to Establish a Registry for Alzheimer’s Disease Word List, n.s. = non-significant, RMSE = Root Mean Squared Error, TMT = Trail Making Test. Education level is categorized into (1) < high school, (2) high school or equivalent, and (3) college and beyond. Reference groups are male and education < high school.

a Tests for which higher scores indicate worse performance.

b TMT-B was completed by 53% of participants.

*p < .05, **p < .01, ***p < .001.

In fact, his actual score was 14, demonstrating below average performance for his demographic group. The RMSE can be incorporated into the equation to obtain the –1.5 SD normative score on CERAD WL Memory for this individual:

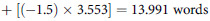




The result shows that the mentioned individual’s CERAD WL Memory score approaches abnormal performance, determined as 1.5 SD below age-, sex- and education-matched individuals. Alternatively, using this individual’s actual score, we can calculate the degree of deviation from the expected score as follows:

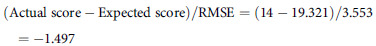




This *z*-score, –1.497, can be converted into its corresponding percentile, i.e., 7^th^. An Excel file to facilitate calculations of norms is provided in the Supplementary Materials and available from the Vietnam Alzheimer’s Disease & Neurocognitive Disorders Association (https://alzvietnam.org/).

### Effects of demographic characteristics

Table 4 presents the effects of age, sex, and education on performance of the neuropsychological battery. Linear regression analyses showed that, on average, 14.6% the variance (adjusted *R*^*2*^) in test performance was explained by demographic variables, ranging from 7% to 29%. Specifically, education explained 0.8% – 8.6%, age explained 3.8% – 14.2%, and sex explained 0.1% – 9.7% of the total variance. Age was consistently and significantly associated with cognitive ability, with younger age relating to better performance on all tests. We found significant effects of sex for measures of memory (CERAD WL Memory: *b* = 2.43, *p* < .001; CERAD WL Recall: *b* = 0.84, *p* < .001; CERAD WL Recognition: *b* = 0.40, *p* = .003) and visuospatial function (Clock Drawing Test: *b* = 0.16, *p* = .043), but not attention (TMT-A: *b* = 1.57, *p* = .59; Digit Span Forward: *b* = –0.02, *p* = .90; Digit Span Backward: *b* = 0.14, *p* = .52), executive function (TMT-B: *b* = 15.36, *p* = .082), nor language (*b* = 0.16, *p* = .043). Specifically, a female advantage was found for all three CERAD WL tasks. In contrast, a male advantage was reported on the Clock Drawing Test. Finally, with the exception of CERAD WL Recognition, education was significantly related to all tests, with a particularly robust effect on CERAD WL Memory, Digit Span Backward, and Animal Naming Test, as indicated by the regression coefficients of different levels of educational attainment.

## Discussion

To our knowledge, this is the first study to generate normative data for a neuropsychological battery in cognitively healthy Vietnamese adults, and to examine the effects of demographic variables on test performance. We found that performance on neuropsychological tests was differentially influenced by age, education, and sex, with age showing the strongest effect. All tests were vulnerable to age, wherein greater age was associated with worse performance, consistent with prior literature documenting that except for general knowledge, the majority of fluid abilities are expected to decrease in later life (Salthouse, 2019). While education exerted significant influence on most test scores, its effect was more robust in some tests than others. Our study coded educational attainment as a categorical variable, and only in the CERAD WL Memory, Digit Span Backward, and Animal Naming Test were consistent differences found across all three levels of education. In CERAD WL Recall, both parts of TMT, Digit Span Forward, and Clock Drawing Test, association of education was less robust, wherein only people with a college degree or beyond outperformed those who did not graduate from high school, but no differences were found between people with or without high school education. The only measure where education did not contribute significantly to performance was CERAD WL Recognition, a finding that is in agreement with previous normative studies on the CERAD neuropsychological battery. One explanation could be overlearning, as participants had to read aloud and memorize the words three times during WL Memory, and were further provided with visual cues during WL Recognition (Liu et al., 2011). This also demonstrated a marked ceiling effect for WL Recognition, such that participants performed relatively well regardless of educational attainment (Kirsebom et al., 2019). In terms of sex, we found a female advantage on verbal learning and recall (all three CERAD WL tasks) and a male advantage in visuospatial perception (Clock Drawing Test)–results that have been well documented in the literature (Karstens et al., 2023).

Several caveats concerning the usage of our normative data in clinical practice should be taken into account. In particular, TMT-B was only completed within the time limit of 300 s by approximately one half of the cohort, presumably by those with good knowledge of the English or French alphabet. Among the remaining half, three-fourths did not complete TMT-B within 300 s and one-fourth failed to finish TMT-B due to several reasons, including not understanding test instructions or not recalling the sequence of the English alphabet. Underperformance on TMT-B was largely explained by lower educational level and more advanced age, comparable to previous reports (Hashimoto et al., 2006; Seo et al., 2006). Hence, the results of TMT-B from this demographic in our study should be interpreted with caution. In the future, we may consider attempts to improve the clinically relevant performance of TMT-B. For example, increasing the permitted time to complete the test (e.g., up to 480 s (Hashimoto et al., 2006) or adding quantitative scores (number of correct lines and errors of commission (Weintraub et al., 2009) can possibly unmask performance variability among individuals and enhance utility in parametric statistical reports (Correia et al., 2015). Notably, even when participants finish the test within the allotted time, the average TMT-B scores of our cohort are considerably lower than those previously reported (Seo et al., 2006; Wang et al., 2011; Weintraub et al., 2018), presumably because of participants’ unfamiliarity with timed assessments and the English alphabet. Hence, TMT-B items could be modified, such that English letters are substituted with Vietnamese letters. That being said, while this adaptation has been realized in multiple languages (e.g., Arabic (Stanczak et al., 2001), Chinese (Lu & Bigler, 2000), Japanese (Hashimoto et al., 2006), and Korean (Seo et al., 2006), it may still have considerable limitations in evaluating older adults, especially those with low educational attainment, as shown in (Hashimoto et al., 2006). Finally, approaches such as removing TMT-B while retaining TMT-A in the battery (Dick et al., 2002) or replacing TMT with its relatively language-free and culturally fair equivalence (e.g, the Color Trails Tests (Dugbartey et al., 2000; McCauley et al., 2023) or TMT-Black and White (Kim et al., 2014) are also feasible.

In addition, a number of distinctions between the application of regression-based norms versus discrete norms are worth mentioning. First, as we did not correct for sex in the discrete norms due to inadequate sample size, normative values of neuropsychological tests known to be affected by this demographic variable may be more accurate using the regression approach. This is particularly evident in CERAD WL Memory; such that given the same age, level of education, and recall performance, a female individual would be expected to correctly remember two items more than a male individual using the regression-based norms, while no sex effect is apparent from the discrete norms table. Second, discrete norms assigned several possible percentiles to a single raw score for some cognitive tests, such as CERAD WL Recognition (Supplementary Table S5) and Clock Drawing Test (Supplementary Table S11), leaving considerable scope for individual interpretation. This finding also demonstrates a more pronounced ceiling effect in the CERAD WL Recognition and Clock Drawing Test than in other measures, i.e., cognitively healthy individuals achieved relatively high scores in these two tests regardless of age, sex, or education. While low ceiling in CERAD WL Recognition was attributable to overlearning as mentioned, for Clock Drawing Test it could be due to our usage of the 5-point Shulman scoring system. Implementing more complex (and time-consuming) scoring methods, e.g., a 10- or 20-point scale, could potentially reduce the skewness of score distribution (Strauss et al., 2006). Additionally, it has been shown that the ceiling effect is diminished and education emerges as a protective factor in the performance of Clock Drawing Test among individuals that developed dementia (Lam et al., 1998). One final distinction between the two norming methods is in the well-documented treatment of age (Kiselica et al., 2024): the regression-based approach treats age as a continuous variable, whereas discrete norming necessitates the creation of artificial age bands. For instance, using discrete norms, a TMT-A score of 100 would correspond to performance between percentiles 7.5^th^ and 15^th^ (>1 SD below norms) for a 59-year-old individual, and between percentiles 15^th^ and 25^th^ for a 60-year-old individual (both with < high school education), indicating an impaired score for the former but not the latter, despite an age gap of only one year. In this case, the artificial age group effect can be avoided by using the regression-based norms calculation.

The strengths of our study include the relatively comprehensive battery of neuropsychological tests that covers most cognitive domains, the translation and adaptation of a verbal memory measure, and generation of both discrete and regression-based norms. However, there are several limitations to consider. The first issue concerns the lack of representation of people with lower educational attainment, largely explained by the data collection taking place in a heavily urbanized city in Vietnam. About 42.0% of the study cohort were college graduates, compared to a percentage of 11.1% of adults having a bachelor’s degree based on a 2020 National Census Bureau Survey, with the majority of these individuals living in either the capital Hanoi (44.8%) or Ho Chi Minh city (38.7%) (Vietnam Department of Population and Labor Statistics, 2019). Similarly, individuals aged 80 – 89 years only accounted for 5.9% of our cohort (*n* = 23). Given that advanced age is associated with a faster rate of cognitive decline and substantial variability in neuropsychological ability, normative values of the 80+ age group may be less accurate compared to those of younger adults and should be interpreted with caution (Arampatzi et al., 2024). Second, neuropsychological measures of participants were collected at a single visit, thus individuals at preclinical stages of neurodegenerative disorders (e.g., dementia) might have been included in the study. As a result, normative standards may be less robust and could overlook the earliest signs of cognitive impairment (Arampatzi et al., 2024; Kapoor et al., 2024). Third, participants were not screened for depressive symptoms. Estimates of the prevalence of depression and anxiety disorders among Vietnamese adults vary across studies, e.g., from 2.6% (Vuong et al., 2011) to 14.2% (Nguyen et al., 2019), warranting assessment of these comorbidities in future norming efforts. Finally, the proportion of variance explained in the regression models averaged only 14.6%, ranging from 7% to 29% for different cognitive tests. This suggests that other than age, sex, and level of education, a variety of demographic (e.g., quality of education and socioeconomic status) and non-demographic factors (e.g., testing environment, participants’ attitude and motivation) could play significant roles in predicting test performance (Kiselica et al., 2024).

In conclusion, we report a relatively comprehensive and sufficiently powered set of normative data for commonly used neuropsychological tests in Vietnamese adults. Administration of the battery can be fully done within 30 minutes, well-suited for clinical appointments. Demographic variables including age and education were associated with performance on all or most measures, while some sex effects consistent with the literature were found. The present study is part of a larger effort to strengthen research capacity and inform policy making in Alzheimer’s disease and related dementia in Vietnam (Vu et al., 2024). Along with many Asian counterparts, Vietnam is experiencing dramatic demographic changes, i.e., a rapidly aging population, leading to a substantial increase in dementia prevalence (Asian Development Bank, 2022; Kosowicz et al., 2023). We specifically hope to improve the interpretation of neuropsychological reports and research, and that testing services will become more widespread throughout the country. To improve the sensitivity and utilization of the battery, further refinements will be necessary, including (i) increasing representation of people with more advanced age and/or lower educational attainment, (ii) incorporating longitudinal assessment to allow for more robust norming, and (iii) applying neuropsychological measures that could be more culturally and linguistically suitable for Vietnamese people.

## Supporting information

Nguyen et al. supplementary material 1 Nguyen et al. supplementary material

Nguyen et al. supplementary material 2 Nguyen et al. supplementary material

## Supplementary material

The supplementary material for this article can be found at https://doi.org/10.1017/S1355617725101100.
