# Supplementary material for: Normative data for Vietnamese population: Effects of age, education, and sex on test performance
Source: J Int Neuropsychol Soc. 2025 Jul 3;31(7-8):573–81. doi: 10.1017/S1355617725101100 (PMC12752475; doi:10.1017/S1355617725101100)
Supplement: Nguyen et al. supplementary material 2 — Nguyen et al. supplementary material [file S1355617725101100sup002.docx]

# **Normative Data for Vietnamese Population: Effects of Age, Education, and Sex on Test Performance**

Truc Tran Thanh Nguyen, Thanh-Liem Do, Huong Thi Thu Tran, Ingo Kilimann, Cong-Thang Tran

**Supplementary Materials**

**Supplementary Methods.** Construction and English equivalents of the Vietnamese translation of CERAD Word List.

**Supplementary Table S1**. Vietnamese version of the CERAD Word List.

**Supplementary Table S2**. Demographic characteristics and mean (SD) test scores of participants who completed and did not complete the Trail Making Test, Part B.

**Supplementary Table S3**. Discrete normative data of CERAD WL Memory.

**Supplementary Table S4**. Discrete normative data of CERAD WL Recall.

**Supplementary Table S5**. Discrete normative data of CERAD WL Recognition.

**Supplementary Table S6**. Discrete normative data of Trail Making Test, Part A.

**Supplementary Table S7**. Discrete normative data of Trail Making Test, Part B.

**Supplementary Table S8**. Discrete normative data of Digit Span Forward.

**Supplementary Table S9**. Discrete normative data of Digit Span Backward.

**Supplementary Table S10**. Discrete normative data of Animal Naming Test.

**Supplementary Table S11**. Discrete normative data of Clock Drawing Test.

**Supplementary Methods.** Construction and English equivalents of the Vietnamese translation of CERAD Word List.

Notably, the majority of Vietnamese nouns consist of either one or two syllables. The *original* Vietnamese version of CERAD Word List consists of 4 single-syllable words and 6 two-syllable words in the Word List Learning (Memory) Task, and 3 single-syllable words and 7 two-syllable words in the foils. The *alternate* version has 4 single-syllable words and 6 two-syllable words in the Word List Learning (Memory) Task; and 3 single-syllable words, 7 two-syllable words in the foils. English equivalents of each word are provided in the following table.

**Supplementary Table S1**. Vietnamese version of the CERAD Word List.

| **Vietnamese Original Word List** | | | | **Vietnamese Alternate Word List** | | | |
| --- | --- | --- | --- | --- | --- | --- | --- |
| **Word** | **No. of syllables** | **Frequency** | **English Equivalent** | **Word** | **No. of syllables** | **Frequency** | **English Equivalent** |
| Hoa* | 1 | AA | Flower | Xe* | 1 | AA | Bike |
| Cây | 1 | AA | Tree | Vườn | 1 | AA | Garden |
| Gạo* | 1 | A | Rice | Cỏ* | 1 | A | Grass |
| Đầu | 1 | AA | Head | Chân | 1 | AA | Leg |
| Mắt* | 1 | AA | Eye | Bụng* | 1 | A | Abdomen |
| Rừng | 1 | AA | Forest | Biển* | 1 | AA | Sea |
| Con gà* | 2 | AA | Chicken | Con chim | 2 | AA | Bird |
| Sư tử | 2 | A | Lion | Cá voi* | 2 | AA | Whale |
| Khách sạn* | 2 | AA | Hotel | Sở thú* | 2 | AA | Zoo |
| Nhà thờ | 2 | A | Church | Ngôi nhà | 2 | A | House |
| Trường học* | 2 | A | School | Thư viện* | 2 | A | Library |
| Ngôi sao* | 2 | A | Star | Mặt trời | 2 | A | Sun |
| Ô tô* | 2 | AA | Car | Máy tính* | 2 | AA | Computer |
| Trang phục | 2 | AA | Uniform | Cà Phê | 2 | AA | Coffee |
| Màu xanh* | 2 | A | Blue (or Green) | Màu vàng | 2 | A | Yellow |
| Em bé | 2 | A | Baby | Con gái* | 2 | A | Girl |
| Bàn tay | 2 | A | Hand | Đôi mắt | 2 | A | Eyes |
| Thác nước | 2 | AA | Waterfall | Hồ bơi | 2 | AA | Swimming pool |
| Bánh mì | 2 | AA | Bread | Mì gói* | 2 | A | Instant noodle |
| Phở* | 1 | A | Pho | Cháo | 1 | A | Porridge |
| **Total** | 33 | AA 11, A 9 |  |  | 33 | AA 10, A 10 |  |

*Note*. AA = word occurs > 50,000 times based on Google search engine for Vietnamese words. A = word occurs 10,000-50,000 times based on Google search engine for Vietnamese words. Phở (pho): a popular Vietnamese soup consisting of broth, rice noodles, herbs and meat.

*: words on the 10-item word list. Words without * are foils used in the Word List Recognition task.

*Reference*

Morris, J. C., Heyman, A., Mohs, R. C., Hughes, J. P., van Belle, G., Fillenbaum, G., Mellits, E. D., & Clark, C. (1989). The Consortium to Establish a Registry for Alzheimer’s Disease (CERAD). Part I. Clinical and neuropsychological assessment of Alzheimer’s disease. *Neurology*, *39*(9), 1159–1165. https://doi.org/10.1212/wnl.39.9.1159

**Supplementary Table S2**. Demographic characteristics and mean (*SD*) test scores of participants who completed and did not complete the Trail Making Test, Part B.

|  | **TMT–B completer**(*n* = 204) | **TMT–B non-completer**(*n* = 181) | ***p* value** | **Effect size** |
| --- | --- | --- | --- | --- |
| ***Demographic*** |  |  |  |  |
| Age, *M* (*SD*) | 58.29 (10.60) | 65.02 (10.28) | <.001 | 0.64 |
| Female, *n* (%) | 119 (58.33) | 101 (55.80) | .69 | 0.02 |
| Education, *n* (%) |  |  | <.001 | 0.42 |
| Less than high school | 18 (8.82) | 67 (37.01) |  |  |
| High school or equivalent | 64 (31.37) | 74 (40.88) |  |  |
| College and beyond | 122 (59.81) | 40 (22.11) |  |  |
| ***Neuropsychological****, M* (*SD*) |  |  |  |  |
| MMSE | 28.14 (1.06) | 27.43 (1.00) | <.001 | –0.68 |
| CERAD WL Memory | 22.49 (3.78) | 19.60 (4.21) | <.001 | –0.72 |
| CERAD WL Recall | 7.48 (1.78) | 6.66 (1.77) | .16 | –0.45 |
| CERAD WL Recognition | 9.31 (1.13) | 8.69 (1.53) | .005 | –0.47 |
| Trail Making Test, Part A^a^ | 58.98 (23.67) | 82.09 (34.94) | <.001 | 0.78 |
| Digit Span Forward | 9.26 (2.03) | 8.40 (2.11) | .24 | –0.41 |
| Digit Span Backward | 6.44 (2.32) | 4.92 (1.91) | <.001 | –0.70 |
| Animal Naming Test | 20.02 (4.66) | 17.86 (4.33) | .14 | –0.47 |
| Clock Drawing Test | 1.45 (0.62) | 1.89 (0.93) | <.001 | 0.56 |
| Neuropsychological test administration time, minutes | 27.00 (4.84) | 30.07 (8.02) | .040 | 0.46 |

*Note*. CERAD WL = Consortium to Establish a Registry for Alzheimer's Disease Word List Tasks; MMSE = Mini-Mental State Examination; TMT–B, Trail Making Test, Part B. Comparisons of neuropsychological measures were adjusted for age, sex, and education. Effect size was calculated as Cohen's *d* or Cramer's *V* where applicable.

# ^a^Missing in 1/204 TMT-B completers (0.4%) and 9/181 TMT-B non-completers (4.9%).

**Supplementary Table S3**. Discrete normative data of CERAD WL Memory.

| **Age group** | **Education** | **Percentile** |  |  |  |  |  |  |
| --- | --- | --- | --- | --- | --- | --- | --- | --- |
|  |  | **3rd (–2 SD)** | **7.5th (–1.5 SD)** | **15th (–1 SD)** | **25th** | **50th** | **75th** | **95th** |
| 40–49 | < HS (*n* = 10) | 16.35 | 18.37 | 20.35 | 21.25 | 24.50 | 27.00 | 28.00 |
|  | HS (*n* = 16) | 17.00 | 17.25 | 19.25 | 20.00 | 22.50 | 25.00 | 30.00 |
|  | ≥ College (*n* = 44) | 18.00 | 18.22 | 21.00 | 22.75 | 25.00 | 27.00 | 28.00 |
| 50–59 | < HS (*n* = 19) | 12.00 | 12.35 | 15.10 | 17.50 | 19.00 | 22.50 | 25.10 |
|  | HS (*n* = 41) | 16.20 | 19.00 | 19.00 | 21.00 | 23.00 | 25.00 | 27.00 |
|  | ≥ College (*n* = 30) | 12.00 | 16.35 | 19.00 | 19.50 | 24.00 | 26.00 | 27.55 |
| 60–69 | < HS (*n* = 28) | 13.00 | 16.00 | 17.00 | 18.75 | 20.00 | 22.00 | 25.30 |
|  | HS (*n* = 50) | 13.47 | 15.00 | 18.00 | 19.00 | 22.50 | 25.00 | 27.00 |
|  | ≥ College (*n* = 51) | 15.50 | 16.75 | 18.00 | 19.00 | 22.00 | 25.00 | 27.00 |
| 70–79 | < HS (*n* = 19) | 11.54 | 12.35 | 13.70 | 15.00 | 16.00 | 20.50 | 22.20 |
|  | HS (*n* = 25) | 10.44 | 12.60 | 14.00 | 15.00 | 19.00 | 21.00 | 24.00 |
|  | ≥ College (*n* = 29) | 13.52 | 14.10 | 16.00 | 18.00 | 20.00 | 23.00 | 24.60 |
| 80–89 | < HS (*n* = 9) | 13.24 | 13.60 | 14.40 | 16.00 | 17.00 | 19.00 | 19.60 |
|  | HS (*n* = 6) | 12.15 | 12.37 | 12.75 | 13.50 | 17.00 | 21.25 | 22.75 |
|  | ≥ College (*n* = 8) | 14.42 | 15.05 | 16.05 | 16.75 | 18.00 | 19.25 | 21.30 |

**Supplementary Table S4**. Discrete normative data of CERAD WL Recall.

| **Age group** | **Education** | **Percentile** |  |  |  |  |  |  |
| --- | --- | --- | --- | --- | --- | --- | --- | --- |
|  |  | **3rd (–2 SD)** | **7.5th (–1.5 SD)** | **15th (–1 SD)** | **25th** | **50th** | **75th** | **95th** |
| 40–49 | < HS (*n* = 10) | 6.27 | 6.67 | 7.00 | 7.25 | 8.00 | 9.75 | 10.00 |
|  | HS (*n* = 16) | 6.00 | 6.12 | 7.25 | 8.00 | 8.00 | 8.00 | 9.25 |
|  | ≥ College (*n* = 44) | 6.00 | 6.22 | 7.00 | 8.00 | 9.00 | 10.00 | 10.00 |
| 50–59 | < HS (*n* = 19) | 2.62 | 4.35 | 5.00 | 6.00 | 7.00 | 8.00 | 8.10 |
|  | HS (*n* = 41) | 3.00 | 5.00 | 5.00 | 6.00 | 8.00 | 9.00 | 10.00 |
|  | ≥ College (*n* = 30) | 6.00 | 6.00 | 6.00 | 7.00 | 7.50 | 8.00 | 10.00 |
| 60–69 | < HS (*n* = 28) | 3.24 | 5.02 | 6.00 | 6.00 | 7.00 | 8.00 | 9.00 |
|  | HS (*n* = 50) | 3.00 | 4.00 | 5.35 | 6.00 | 7.00 | 8.00 | 10.00 |
|  | ≥ College (*n* = 51) | 4.00 | 4.00 | 5.00 | 6.00 | 8.00 | 8.00 | 9.00 |
| 70–79 | < HS (*n* = 19) | 3.54 | 4.35 | 5.00 | 5.50 | 7.00 | 8.00 | 9.10 |
|  | HS (*n* = 25) | 3.16 | 4.00 | 4.60 | 5.00 | 7.00 | 7.00 | 8.00 |
|  | ≥ College (*n* = 29) | 2.84 | 4.00 | 5.00 | 5.00 | 7.00 | 8.00 | 9.00 |
| 80–89 | < HS (*n* = 9) | 1.96 | 3.40 | 5.00 | 5.00 | 6.00 | 6.00 | 7.00 |
|  | HS (*n* = 6) | 2.30 | 2.75 | 3.50 | 4.25 | 5.00 | 5.75 | 7.50 |
|  | ≥ College (*n* = 8) | 4.21 | 4.52 | 5.00 | 5.00 | 5.00 | 6.25 | 7.65 |

**Supplementary Table S5**. Discrete normative data of CERAD WL Recognition.

| **Age group** | **Education** | **Percentile** |  |  |  |  |  |  |
| --- | --- | --- | --- | --- | --- | --- | --- | --- |
|  |  | **3rd (–2 SD)** | **7.5th (–1.5 SD)** | **15th (–1 SD)** | **25th** | **50th** | **75th** | **95th** |
| 40–49 | < HS (*n* = 10) | 8.27 | 8.67 | 9.00 | 9.25 | 10.00 | 10.00 | 10.00 |
|  | HS (*n* = 16) | 8.45 | 9.00 | 9.00 | 9.00 | 10.00 | 10.00 | 10.00 |
|  | ≥ College (*n* = 44) | 7.58 | 9.00 | 9.00 | 10.00 | 10.00 | 10.00 | 10.00 |
| 50–59 | < HS (*n* = 19) | 7.00 | 7.35 | 8.00 | 8.00 | 9.00 | 10.00 | 10.00 |
|  | HS (*n* = 41) | 8.00 | 8.00 | 8.00 | 9.00 | 10.00 | 10.00 | 10.00 |
|  | ≥ College (*n* = 30) | 5.00 | 8.00 | 8.00 | 8.25 | 10.00 | 10.00 | 10.00 |
| 60–69 | < HS (*n* = 28) | 5.62 | 6.00 | 6.05 | 8.00 | 9.50 | 10.00 | 10.00 |
|  | HS (*n* = 50) | 6.00 | 6.00 | 8.00 | 9.00 | 10.00 | 10.00 | 10.00 |
|  | ≥ College (*n* = 51) | 7.00 | 7.00 | 8.00 | 8.00 | 9.00 | 10.00 | 10.00 |
| 70–79 | < HS (*n* = 19) | 5.00 | 5.70 | 7.00 | 8.00 | 9.00 | 10.00 | 10.00 |
|  | HS (*n* = 25) | 3.72 | 5.60 | 6.60 | 7.00 | 9.00 | 9.00 | 10.00 |
|  | ≥ College (*n* = 29) | 5.84 | 7.00 | 7.20 | 8.00 | 9.00 | 10.00 | 10.00 |
| 80–89 | < HS (*n* = 9) | 3.96 | 5.40 | 7.20 | 8.00 | 9.00 | 9.00 | 10.00 |
|  | HS (*n* = 6) | 3.60 | 4.50 | 6.00 | 7.25 | 8.50 | 9.75 | 10.00 |
|  | ≥ College (*n* = 8) | 8.21 | 8.52 | 9.00 | 9.00 | 9.00 | 9.25 | 10.00 |

**Supplementary Table S6**. Discrete normative data of Trail Making Test, Part A.

| **Age group** | **Education** | **Percentile** |  |  |  |  |  |  |
| --- | --- | --- | --- | --- | --- | --- | --- | --- |
|  |  | **3rd (–2 SD)** | **7.5th (–1.5 SD)** | **15th (–1 SD)** | **25th** | **50th** | **75th** | **95th** |
| 40–49 | < HS (*n* = 10) | 134.88 | 112.20 | 92.95 | 87.00 | 61.50 | 59.25 | 44.00 |
|  | HS (*n* = 15) | 112.44 | 101.65 | 94.30 | 80.50 | 60.00 | 56.50 | 28.40 |
|  | ≥ College (*n* = 44) | 61.42 | 60.00 | 56.10 | 52.25 | 41.50 | 33.00 | 23.15 |
| 50–59 | < HS (*n* = 18) | 169.80 | 142.40 | 92.70 | 81.00 | 60.00 | 51.50 | 40.10 |
|  | HS (*n* = 41) | 121.60 | 111.00 | 90.00 | 83.00 | 63.00 | 50.00 | 35.00 |
|  | ≥ College (*n* = 30) | 73.21 | 69.12 | 62.00 | 61.75 | 51.00 | 40.00 | 28.35 |
| 60–69 | < HS (*n* = 26) | 156.00 | 130.50 | 120.00 | 88.75 | 69.00 | 55.25 | 35.00 |
|  | HS (*n* = 50) | 132.66 | 115.95 | 92.60 | 84.75 | 60.00 | 53.25 | 41.35 |
|  | ≥ College (*n* = 51) | 117.00 | 101.00 | 92.00 | 79.00 | 60.00 | 50.00 | 36.50 |
| 70–79 | < HS (*n* = 18) | 121.47 | 120.00 | 116.70 | 100.00 | 84.50 | 62.50 | 42.10 |
|  | HS (*n* = 24) | 161.72 | 144.95 | 125.40 | 120.00 | 86.00 | 58.00 | 43.20 |
|  | ≥ College (*n* = 29) | 130.32 | 104.90 | 98.00 | 80.00 | 60.00 | 50.00 | 42.00 |
| 80–89 | < HS (*n* = 7) | 168.20 | 165.50 | 161.00 | 157.50 | 142.00 | 136.00 | 115.80 |
|  | HS (*n* = 5) | 169.00 | 160.00 | 145.00 | 125.00 | 89.00 | 80.00 | 72.00 |
|  | ≥ College (*n* = 7) | 116.40 | 111.00 | 102.00 | 98.00 | 88.00 | 70.50 | 69.30 |

**Supplementary Table S7**. Discrete normative data of Trail Making Test, Part B.

| **Age group** | **Education** | **Percentile** |  |  |  |  |  |  |
| --- | --- | --- | --- | --- | --- | --- | --- | --- |
|  |  | **3rd (–2 SD)** | **7.5th (–1.5 SD)** | **15th (–1 SD)** | **25th** | **50th** | **75th** | **95th** |
| 40–49 | < HS (*n* = 5) | 262.32 | 259.80 | 255.60 | 250.00 | 210.00 | 177.00 | 125.80 |
|  | HS (*n* = 8) | 280.93 | 259.82 | 226.35 | 203.25 | 145.00 | 120.00 | 113.50 |
|  | ≥ College (*n* = 40) | 198.13 | 187.15 | 154.05 | 142.75 | 92.00 | 73.50 | 54.45 |
| 50–59 | < HS (*n* = 6) | 222.40 | 217.00 | 208.00 | 201.50 | 196.00 | 183.00 | 136.50 |
|  | HS (*n* = 25) | 274.00 | 260.00 | 248.80 | 220.00 | 140.00 | 108.00 | 68.80 |
|  | ≥ College (*n* = 24) | 233.78 | 206.05 | 196.85 | 161.50 | 115.00 | 99.50 | 66.60 |
| 60–69 | < HS (*n* = 4) | 159.94 | 155.35 | 147.70 | 137.50 | 124.50 | 118.75 | 115.75 |
|  | HS (*n* = 26) | 280.50 | 261.75 | 238.50 | 197.50 | 160.50 | 121.00 | 113.25 |
|  | ≥ College (*n* = 35) | 279.74 | 258.75 | 200.00 | 193.50 | 140.00 | 100.00 | 63.50 |
| 70–79 | < HS (*n* = 2) | 179.67 | 179.17 | 178.35 | 177.25 | 174.50 | 171.75 | 169.55 |
|  | HS (*n* = 3) | 183.66 | 180.15 | 174.30 | 166.50 | 147.00 | 118.50 | 95.70 |
|  | ≥ College (*n* = 20) | 300.00 | 292.77 | 258.35 | 230.50 | 177.50 | 145.25 | 121.90 |
| 80–89 | < HS (*n* = 1) | . | . | . | . | 298.00 | . | . |
|  | HS (*n* = 2) | 231.34 | 230.35 | 228.70 | 226.50 | 221.00 | 215.50 | 211.10 |
|  | ≥ College (*n* = 3) | 272.80 | 262.00 | 244.00 | 220.00 | 160.00 | 140.00 | 124.00 |

**Supplementary Table S8**. Discrete normative data of Digit Span Forward.

| **Age group** | **Education** | **Percentile** |  |  |  |  |  |  |
| --- | --- | --- | --- | --- | --- | --- | --- | --- |
|  |  | **3rd (–2 SD)** | **7.5th (–1.5 SD)** | **15th (–1 SD)** | **25th** | **50th** | **75th** | **95th** |
| 40–49 | < HS (*n* = 10) | 5.27 | 5.67 | 6.70 | 8.25 | 9.50 | 10.75 | 11.55 |
|  | HS (*n* = 16) | 2.70 | 6.12 | 7.25 | 8.00 | 10.00 | 11.00 | 12.00 |
|  | ≥ College (*n* = 44) | 6.29 | 7.22 | 8.45 | 9.75 | 10.00 | 12.00 | 12.00 |
| 50–59 | < HS (*n* = 19) | 6.00 | 6.35 | 7.00 | 8.00 | 9.00 | 10.50 | 12.00 |
|  | HS (*n* = 41) | 6.00 | 7.00 | 7.00 | 8.00 | 10.00 | 11.00 | 12.00 |
|  | ≥ College (*n* = 30) | 6.00 | 7.00 | 8.00 | 8.00 | 9.50 | 11.00 | 11.55 |
| 60–69 | < HS (*n* = 28) | 4.00 | 5.02 | 6.00 | 6.75 | 7.00 | 8.25 | 11.00 |
|  | HS (*n* = 50) | 5.00 | 6.00 | 6.00 | 7.00 | 9.00 | 10.75 | 12.00 |
|  | ≥ College (*n* = 51) | 6.00 | 6.75 | 7.50 | 8.00 | 9.00 | 11.00 | 12.00 |
| 70–79 | < HS (*n* = 19) | 4.08 | 5.00 | 5.00 | 5.50 | 6.00 | 9.00 | 11.10 |
|  | HS (*n* = 25) | 4.72 | 5.00 | 6.00 | 6.00 | 7.00 | 9.00 | 10.80 |
|  | ≥ College (*n* = 29) | 5.00 | 6.00 | 6.00 | 7.00 | 8.00 | 10.00 | 11.60 |
| 80–89 | < HS (*n* = 9) | 6.00 | 6.00 | 6.40 | 8.00 | 9.00 | 10.00 | 10.60 |
|  | HS (*n* = 6) | 6.15 | 6.37 | 6.75 | 7.25 | 8.50 | 9.00 | 11.25 |
|  | ≥ College (*n* = 8) | 6.00 | 6.00 | 6.15 | 8.25 | 9.00 | 10.00 | 10.00 |

**Supplementary Table S9**. Discrete normative data of Digit Span Backward.

| **Age group** | **Education** | **Percentile** |  |  |  |  |  |  |
| --- | --- | --- | --- | --- | --- | --- | --- | --- |
|  |  | **3rd (–2 SD)** | **7.5th (–1.5 SD)** | **15th (–1 SD)** | **25th** | **50th** | **75th** | **95th** |
| 40–49 | < HS (*n* = 10) | 2.27 | 2.67 | 3.00 | 3.50 | 6.00 | 6.75 | 8.55 |
|  | HS (*n* = 16) | 3.45 | 4.00 | 4.00 | 4.75 | 6.00 | 6.25 | 7.00 |
|  | ≥ College (*n* = 44) | 3.29 | 4.00 | 4.00 | 5.00 | 7.00 | 9.00 | 11.00 |
| 50–59 | < HS (*n* = 19) | 2.00 | 2.00 | 3.40 | 4.00 | 4.00 | 5.50 | 7.20 |
|  | HS (*n* = 41) | 3.00 | 3.00 | 3.00 | 4.00 | 6.00 | 8.00 | 10.00 |
|  | ≥ College (*n* = 30) | 2.00 | 4.00 | 4.00 | 5.00 | 6.50 | 8.00 | 11.00 |
| 60–69 | < HS (*n* = 28) | 2.81 | 3.00 | 3.05 | 4.00 | 5.00 | 6.00 | 7.65 |
|  | HS (*n* = 50) | 3.00 | 3.00 | 4.00 | 4.00 | 5.00 | 7.00 | 10.00 |
|  | ≥ College (*n* = 51) | 2.00 | 3.00 | 4.00 | 4.00 | 6.00 | 8.00 | 10.00 |
| 70–79 | < HS (*n* = 19) | 2.54 | 3.00 | 3.00 | 4.00 | 4.00 | 5.50 | 7.10 |
|  | HS (*n* = 25) | 2.00 | 2.80 | 3.00 | 3.00 | 5.00 | 6.00 | 8.60 |
|  | ≥ College (*n* = 29) | 3.00 | 3.10 | 4.00 | 4.00 | 6.00 | 6.00 | 8.60 |
| 80–89 | < HS (*n* = 9) | 2.24 | 2.60 | 3.20 | 4.00 | 4.00 | 4.00 | 6.20 |
|  | HS (*n* = 6) | 3.00 | 3.00 | 3.00 | 3.50 | 5.50 | 6.75 | 8.50 |
|  | ≥ College (*n* = 8) | 2.00 | 2.00 | 2.05 | 2.75 | 4.00 | 4.00 | 4.00 |

**Supplementary Table S10**. Discrete normative data of Animal Naming.

| **Age group** | **Education** | **Percentile** |  |  |  |  |  |  |
| --- | --- | --- | --- | --- | --- | --- | --- | --- |
|  |  | **3rd (–2 SD)** | **7.5th (–1.5 SD)** | **15th (–1 SD)** | **25th** | **50th** | **75th** | **95th** |
| 40–49 | < HS (*n* = 10) | 12.54 | 13.35 | 14.35 | 15.75 | 20.50 | 22.75 | 23.55 |
|  | HS (*n* = 16) | 11.80 | 14.25 | 16.25 | 17.75 | 20.00 | 23.25 | 26.25 |
|  | ≥ College (*n* = 43) | 17.26 | 18.00 | 19.00 | 20.00 | 22.00 | 26.00 | 27.90 |
| 50–59 | < HS (*n* = 19) | 10.54 | 11.35 | 12.00 | 14.50 | 16.00 | 19.50 | 23.20 |
|  | HS (*n* = 41) | 13.00 | 14.00 | 15.00 | 16.00 | 20.00 | 22.00 | 24.00 |
|  | ≥ College (*n* = 30) | 12.35 | 14.35 | 16.35 | 18.25 | 21.00 | 23.00 | 25.55 |
| 60–69 | < HS (*n* = 28) | 9.81 | 11.05 | 14.00 | 14.75 | 16.50 | 20.25 | 25.00 |
|  | HS (*n* = 50) | 11.00 | 11.67 | 13.35 | 15.00 | 18.50 | 22.00 | 25.00 |
|  | ≥ College (*n* = 51) | 11.00 | 12.75 | 15.00 | 18.00 | 21.00 | 23.00 | 26.50 |
| 70–79 | < HS (*n* = 18) | 11.02 | 12.27 | 13.00 | 13.25 | 18.00 | 18.75 | 20.90 |
|  | HS (*n* = 25) | 10.72 | 12.60 | 13.60 | 14.00 | 17.00 | 20.00 | 26.20 |
|  | ≥ College (*n* = 29) | 10.00 | 11.20 | 14.00 | 15.00 | 17.00 | 24.00 | 26.20 |
| 80–89 | < HS (*n* = 9) | 9.96 | 11.40 | 13.20 | 14.00 | 15.00 | 18.00 | 21.20 |
|  | HS (*n* = 6) | 12.60 | 13.50 | 15.00 | 16.00 | 16.00 | 17.50 | 22.50 |
|  | ≥ College (*n* = 8) | 13.21 | 13.52 | 14.00 | 14.00 | 15.50 | 17.50 | 19.65 |

**Supplementary Table S11**. Discrete normative data of Clock Drawing Test.

| **Age group** | **Education** | **Percentile** |  |  |  |  |  |  |
| --- | --- | --- | --- | --- | --- | --- | --- | --- |
|  |  | **3rd (–2 SD)** | **7.5th (–1.5 SD)** | **15th (–1 SD)** | **25th** | **50th** | **75th** | **95th** |
| 40–49 | < HS (*n* = 10) | 2.00 | 2.00 | 2.00 | 1.75 | 1.00 | 1.00 | 1.00 |
|  | HS (*n* = 16) | 3.00 | 2.87 | 2.00 | 2.00 | 1.00 | 1.00 | 1.00 |
|  | ≥ College (*n* = 44) | 2.00 | 2.00 | 2.00 | 2.00 | 1.00 | 1.00 | 1.00 |
| 50–59 | < HS (*n* = 18) | 4.00 | 3.72 | 2.45 | 2.00 | 2.00 | 2.00 | 1.00 |
|  | HS (*n* = 41) | 3.00 | 2.00 | 2.00 | 2.00 | 2.00 | 1.00 | 1.00 |
|  | ≥ College (*n* = 30) | 2.00 | 2.00 | 2.00 | 2.00 | 1.00 | 1.00 | 1.00 |
| 60–69 | < HS (*n* = 27) | 4.00 | 3.05 | 2.10 | 2.00 | 2.00 | 1.00 | 1.00 |
|  | HS (*n* = 50) | 4.00 | 3.00 | 2.65 | 2.00 | 2.00 | 1.00 | 1.00 |
|  | ≥ College (*n* = 51) | 3.00 | 2.25 | 2.00 | 2.00 | 1.00 | 1.00 | 1.00 |
| 70–79 | < HS (*n* = 18) | 3.49 | 2.72 | 2.00 | 2.00 | 2.00 | 1.00 | 1.00 |
|  | HS (*n* = 25) | 5.00 | 4.20 | 3.00 | 2.00 | 2.00 | 1.00 | 1.00 |
|  | ≥ College (*n* = 29) | 2.16 | 2.00 | 2.00 | 2.00 | 1.00 | 1.00 | 1.00 |
| 80–89 | < HS (*n* = 9) | 4.76 | 4.40 | 4.00 | 4.00 | 3.00 | 2.00 | 1.40 |
|  | HS (*n* = 6) | 3.85 | 3.62 | 3.25 | 2.75 | 2.00 | 1.25 | 1.00 |
|  | ≥ College (*n* = 8) | 3.58 | 2.95 | 2.00 | 2.00 | 2.00 | 2.00 | 1.35 |
